# Case-control study of sudden infant death syndrome in Lithuania, 1997–2000

**DOI:** 10.1186/1471-2431-5-41

**Published:** 2005-11-13

**Authors:** Vilija Bubnaitienė, Ramunė Kalėdienė, Rimantas Kėvalas

**Affiliations:** 1 Department of Pediatrics, Kaunas University of Medicine, Eiveniu 2, 5009 Kaunas, Lithuania; 2 Department of Public Health, Kaunas University of Medicine, Eiveniu 4, 5009 Kaunas, Lithuania

## Abstract

**Background:**

To identify risk factors for sudden infant death syndrome relevant in Lithuania.

**Methods:**

A nationwide case-control study surveying parents of 35 infants who died from sudden infant death syndrome during the period of 1997–2000 and parents of 145 control infants matched with SIDS infants for date of birth and for region of birth was carried out.

**Results:**

Deaths incidence was greater in the warm period (60%) *vs. *cold period (40%). Prone and side sleeping positions both carried no increased risk of sudden infant death syndrome compared with supine because of a rare prone sleeping (4.1% of controls *vs. *0% of dead infants) and more prevalent side than supine sleeping (84.8% of controls *vs. *94.3% of dead infants) in the controls as well as the cases. Bed sharing for the whole night as a risk factor for sudden infant death syndrome has not been confirmed, either, as bed sharing was common only for the controls (13.8% of controls *vs. *0% of dead infants). Routine sleeping environment factors such as heavy wrapping (≥4 togs) of an infant (odds ratio 8.49; 95% confidence interval 2.38 to 30.32), sleeping in a bassinet (4.22; 1.16 to 15.38) and maternal factors such as maternal education ≤12 years (4.48; 1.34 to 14.94), unplanned pregnancy (5.22; 1.49 to 18.18) and ≥2 previous live births (3.90; 1.00 to 15.10) were significantly associated with sudden infant death syndrome on multivariate analysis.

**Conclusion:**

The results of this first population-based case-control study have shed some light on the epidemiology of the syndrome in Lithuania. Although the mortality of sudden infant death syndrome in Lithuania is not high, it might be lowered moreover by public informing about sudden infant death syndrome and related risk factors. Special attention must be paid to mothers with low education on potentially modifiable risk factors such as routine heavy wrapping of an infant during sleep, routine sleeping in a bassinet and unplanned pregnancy.

## Background

Despite the fact that sudden infant death syndrome (SIDS) is the leading cause of postneonatal infant mortality in most developed countries, SIDS incidence varies greatly in different countries and between regions within countries worldwide. Before 1990–1991, SIDS incidence varied from 1 to 6 cases per 1000 live births [1]. Since 1991 SIDS incidence has declined significantly in a lot of countries and now varies from 0.1 to 1.5 cases per 1000 live births [2]. Unfortunately, the cause of SIDS and variations of incidence in different countries remain unclear. In recent years, there has been considerable interest in the role of infant care practices and sleeping environment in SIDS. Sleeping prone has been found to be an especially strong and consistent risk factor across different societies and countries, and modification of this practice has been associated with a major reduction in SIDS incidence [3,4]. In the meantime the average mortality rate from SIDS in Lithuania during the period of 1997–2000 was 0.3 per 1000 live births and was low, if compared to international mortality rates though no risks reducing campaign has been performed.

As considerable differences of SIDS risk factors importance may exist in different countries, the aim of our study was to identify factors associated with and predicting increased risk of SIDS relevant in Lithuania.

## Materials and methods

### Study design and subjects

The survey was performed as a retrospective case-control study during the period of 2002–2003. The main case-control study instruments were questionnaires for cases and controls. The questionnaire for cases consisted of 89 standardized questions concerning infant death circumstances, demographic factors, routine practices in sleeping environment, infant and maternal medical history, parental socioeconomic status and lifestyle. The questionnaire for controls consisted of 81 standardized questions concerning demographic factors, routine practices in sleeping environment, infant and maternal medical history, parental socioeconomic status and lifestyle. No reference sleep was assigned for the control group as the time frame was too long. Questions were of a "yes" or "no" or multiple choice nature or needed data in figures, such as birth date, birth weight, death time, and others.

Questionnaires for cases were completed by the research interviewer during a home visit. Dialogue about aims of visit took the priority. Afterward standardized questions for SIDS cases were asked. SIDS parents were questioned only on a receipt of underwritten consent of participation in the study. The mean time between SIDS death and completing the questionnaire was 3.9 ± 0.2 years. Questionnaires for controls together with a sheet of information and consent of participation in the study were distributed by mail to control parents.

Data of SIDS infants were obtained from computerized database of Lithuanian Department of Statistics. Initially all of 45 infants whose death was attributed to sudden infant death syndrome (ICD-10 – R 95 and R 96) during the period of 1997–2000 were included in the study. Data of control infants were obtained from Lithuanian Medical Birth Registry. Initially 225 controls matched with SIDS infants for date of birth within one month and for region of birth were picked randomly. Selection results were rectified according to Lithuanian Infant Mortality Registry data and 3 infants who died during the period of 1997–2000 were excluded from the control sample.

### Statistical analysis

Data of the study were processed using STATISTIKA/w 5 and SPSS/w 10 (*Statistical Package for Social Science*) software.

Student t and Fisher's (exact) criteria were used for comparison of means. As using these criteria is possible only when sample variables are distributed normally, Kolmogorov-Smirnov goodness-of-fit test was performed in order to assess the normality of distribution.

Chi-square and logistic regression analyses were used to examine differences in the prevalence and prediction of various sleeping environment, maternal, infant, parental socioeconomic and lifestyle risk factors among SIDS cases and controls. Fisher's exact test was utilized for small cell frequencies. Variables significant on univariate analysis were included in multivariate analysis using a conditional logistic regression. Later on, multivariate models were constructed with the backward stepwise procedure for variables significant at the 5% level. The estimations of both univariate and multivariate analysis resulted in odds ratio (OR) with their 95% confidence intervals (CI).

The hypotheses were considered statistically significant at the level of p < 0.05, very significant at the level of p < 0.01 and especially significant at the level of p < 0.001. The hypotheses were considered statistically not significant at the level of p > 0.05.

## Results

### Response cases and controls

10 SIDS cases (22.2%) of the total 45 were excluded from the analysis: 5 because the families could not be traced, having moved from the notified inhabitation place; 2 because the families refused an interview and 3 because they fell short of accepted SIDS definition criteria after review of the case history on the grounds of an interview. We defined SIDS according to Willinger as the sudden death of an infant aged from 7 to 365 days, which remained unexplained after performance of a complete postmortem investigation, including an autopsy, examination of the death scene, and review of the case history [5]. Of the potential 222 control families 40 were not available, 20 refused an interview and 17 were rejected.

Finally, data of 35 SIDS cases and 145 controls (1:4) have entered the final statistical analysis.

### Deaths of sudden infant death syndrome: relation to age, season and time of death

**Figure 1:**
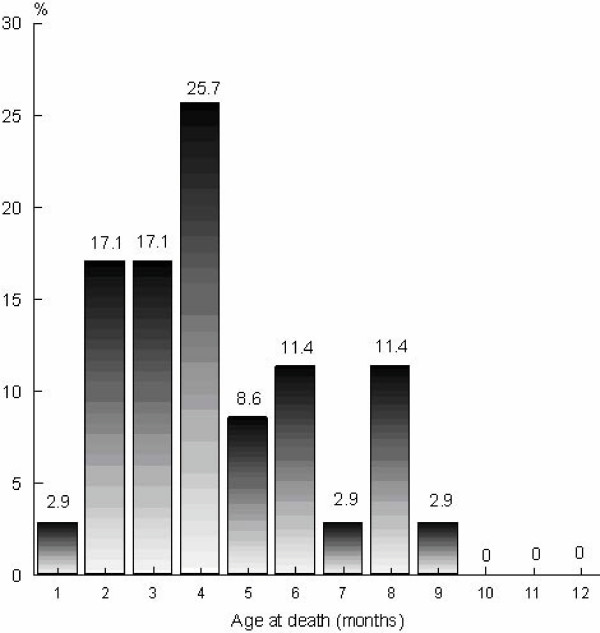
Age at death in months of SIDS infants (n = 35).

The mean (± SE) age at death in SIDS infants was 114.1 ± 9.6 days and varied from 23 to 235 days. A χ^2 ^test of the null hypothesis of a uniform frequency distribution over 12 months was significant (*χ*^*2 *^= *15.5*; *df *= *11*; *p *<*0.05*). The proportion of SIDS deaths was greater in the first half-year of age (82.8%) than in the second half-year (17.2%) with a peak incidence from 2 to 4 month of age (59.9%) (Figure 1).

**Figure 2:**
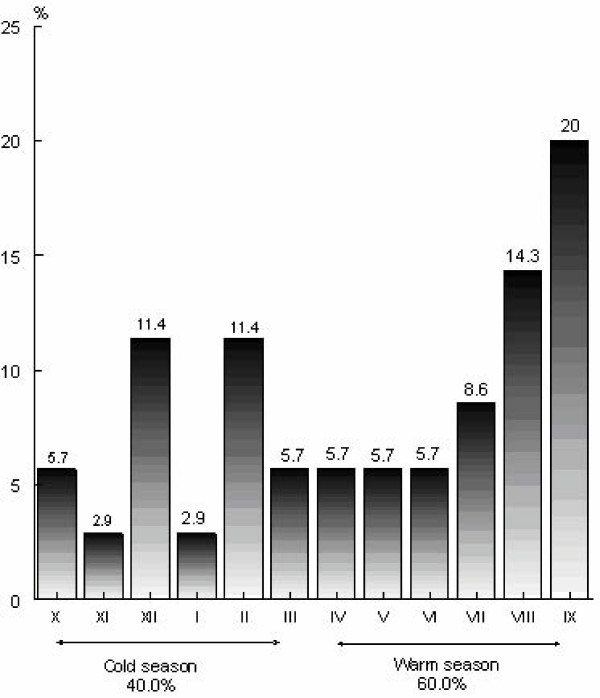
Seasonal distribution of SIDS infants (n = 35).

SIDS deaths according to season were classified into two periods – cold period (six lowest temperature months – October to March) and warm period (six highest temperature months – April to September) (Figure 2). SIDS deaths incidence was greater in the warm period (60%) *vs. *cold period (40%).

Most of the SIDS deaths occurred during what the parents classified as night sleep. 74.3% of infants (26/35) were discovered dead between 1.50 and 9.00 hours and 25.7% (9/35) – between 9.30 and 14.30 hours. The interval between the time that infants were last seen or heard alive and the time found dead was 186.2 ± 24.6 min.

### Univariate results

A univariate analysis was carried out to estimate the strength of the relationship between SIDS and factors concerning routine practices in sleeping environment, infant and maternal medical history, parental socioeconomic status and lifestyle. Within view of SIDS mothers reluctance to report alcohol consumption, especially frequency of alcohol consumption, this variable had not been involved into univariate analysis. Frequencies, odds ratios and p values of all the univariate findings are presented in Table 1. Fisher's exact test was utilized for variables with small cell frequencies. The baseline comparison group always had the opposite definition, for example, infant birth weight <2500.0 g was compared with infant birth weight ≥2500.0 g, unless otherwise stated in the table.

**Table 1:** Univariate findings of sudden infant death syndrome

**Variables**	**Proportion (%) of:**		**Odds ratio (95% CI)**	**p**
			
	**Cases**	**Controls**		
SLEEPING ENVIRONMENT FACTORS*				
Position when put down to sleep:				
• supine	2/35 (5.7)	16/145 (11.0)	1.00 (reference)	
• side	33/35 (94.3)	123/145 (84.8)	2.15 [0.47; 9.80]	0.325
• prone	0/35 (0)	6/145 (4.1)	0.00	0.999
Sleeping surface:				
• crib	15/35 (42.9)	113/145 (77.9)	1.00 (reference)	
• bassinet	19/35 (54.3)	16/145 (11.0)	8.95 [3.80; 21.05]	<0.001
• full-sized bed (parental or sofa)	1/35 (2.9)	16/145 (11.0)	0.47 [0.05; 3.81]	0.480
Substandard infant mattress**	25/35 (71.4)	66/145 (45.5)	3.09 [1.38; 6.89]	0.006
Waterproof cloth over the mattress	18/35 (51.4)	45/145 (31.0)	2.35 [1.11; 4.98]	0.025
Use of a pillow^#^	32/35 (91.4)	135/145 (93.1)	n/a	0.268
Wearing ≥4 togs	24/35 (68.6)	31/145 (21.4)	8.02 [3.55; 18.16]	<0.001
Wearing a cap	17/35 (48.6)	24/145 (16.6)	4.76 [2.15; 10.54]	<0.001
Type of bedding:				
• medium warmth type	18/35 (57.1)	62/145 (42.7)	1.00 (reference)	
• warm type	14/35 (40.0)	80/145 (55.2)	0.54 [0.25; 1.16]	0.114
• unknown type	1/35 (2.9)	3/145 (2.1)	1.03 [0.10; 10.50]	0.978
Bed sharing with parent(s) for the whole night ^#^	0/35 (0)	20/145 (13.8)	n/a	0.010
Sleeping in a room alone^#^	32/35 (91.4)	135/145 (93.1)	n/a	0.268
INFANT FACTORS				
Male sex	23/35 (65.7)	86/145 (59.3)	1.32 [0.61; 2.85]	0.487
Birth weight < 2500.0 g^#^	5/35 (14.3)	2/145 (1.4)	n/a	0.003
Gestation ≤36 weeks^#^	3/35 (8.6)	2/145 (1.4)	n/a	0.051
Any neonatal problem^#^	3/35 (8.6)	12/145 (8.3)	n/a	0.589
Any congenital anomaly^#^	2/35 (5.7)	12/145 (8.3)	n/a	0.463
Twins birth^#^	0/35 (0)	1/145 (0.7)	n/a	0.810
Bottle feeding of the birth	7/35 (20.0)	7/144 (4.9)	4.89 [1.59; 15.05]	0.006
No dummy when sleeping	18/35 (51.4)	57/145 (39.3)	1.63 [0.78; 3.43]	0.194
MATERNAL FACTORS				
Unplanned pregnancy	30/35 (85.7)	59/145 (40.7)	8.75 [3.21; 23.85]	<0.001
Late (month 4–9) or no prenatal care	16/35 (45.7)	24/144 (16.7)	4.25 [1.91; 9.41]	<0.001
Previous stillbirth^#^	2/35 (5.7)	18/144 (12.5)	n/a	0.204
Previous interruption of pregnancy^#^	3/35 (8.6)	12/145 (8.3)	n/a	0.589
Previous infant death^#^	1/35 (2.9)	4/144 (2.8)	n/a	0.668
≥2 previous live births	26/35 (74.3)	74/144 (51.4)	2.73 [1.19; 6.24]	0.017
SOCIOECONOMIC FACTORS				
Maternal age ≤20 y or ≥35 y	15/35 (42.9)	24/145 (16.6)	3.78 [1.69; 8.41]	0.001
Paternal age ≤20 y or ≥35 y	12/33 (36.4)	21/135 (15.6)	3.10 [1.33; 7.25]	0.009
Maternal education ≤12 y	25/35 (71.4)	43/145 (29.7)	5.93 [2.62; 13.40]	<0.001
Paternal education ≤12 y	19/33 (57.6)	56/143 (39.2)	2.11 [0.98; 4.54]	0.057
Single mother***	7/35 (20.0)	11/145 (7.6)	3.04 [1.09; 8.54]	0.034
No waged income	9/33 (27.3)	15/141 (10.6)	3.15 [1.24; 8.02]	0.016
Renting or living with parents	16/35 (45.7)	70/145 (48.3)	0.90 [0.43; 1.89]	0.785
Worse than fair housing conditions^#^	10/35 (28.6)	4/144 (2.8)	n/a	<0.001
Overcrowding**** ^#^	32/35 (91.4)	110/144 (76.4)	n/a	0.035
Worse than fair self-perceived economic family status	25/35 (71.4)	16/145 (11.0)	20.16 [8.21; 49.51]	<0.001
PARENTAL LIFESTYLE FACTORS				
Maternal smoking during pregnancy	10/35 (28.6)	8/145 (5.5)	6.85 [2.46; 19.05]	<0.001
Paternal smoking during pregnancy	26/35 (74.3)	73/145 (50.3)	2.85 [1.25; 6.50]	0.013
Exposure to smoking during pregnancy (one or both of parents smoked)	28/35 (80.0)	73/145 (50.3)	3.94 [1.62; 9.61]	0.003

*Variables are for routine practices.

**Includes pallet, twisted plaid, pillow

***Includes mothers who were divorced, widowed, unmarried and living separately from the father of infant.

****Living >1 person per room.

^#^Fisher's exact test is carried out, hence odds ratio not applicable (n/a).

Over 30 factors were analyzed, and more than half of them were significant. Factors significant on univariate analysis were routine sleeping in a bassinet, routine use of substandard infant mattresses, such as pallet, twisted plaid or pillow for sleep, routine use of waterproof cloth over the mattress for sleep, routine wearing of ≥4 togs during sleep, routine wearing of cap during sleep, routine bed sharing with parent(s) for the whole night, birth weight <2500.0 g, gestation ≤36 weeks, bottle feeding off the birth, unplanned pregnancy, late (initial prenatal visit in month 4–9 of pregnancy) or no prenatal care, ≥2 previous live births, maternal age ≤20 or ≥35 years, paternal age ≤20 or ≥35 years, maternal education ≤12 years, single mother (those who were divorced, widowed, unmarried and living separately from the father of an infant), households without waged income, worse than fair housing conditions, worse than fair self-perceived economic family status, overcrowding (>1 person per room), maternal smoking during pregnancy, paternal smoking during pregnancy, and exposure to smoking during pregnancy when just one or both of the parents smoked.

Examples of factors that were not significant on univariate analysis included routine sleeping positions, routine sleeping in a full-sized bed (parental bed or sofa/couch), routinely used bedding type, routine use of pillow, routine sleeping in room alone, male sex, any neonatal problem, any congenital problem, twin birth, no dummy when sleeping, previous stillbirth, previous interruption of pregnancy, previous infant death, paternal education ≤12 years, and households renting or living with parents.

### Multivariate results

A multivariate analysis was carried out to determine which factors were independently significant when controlled for other factors found to be important in the study. In the course of correlation analysis some correlates have been excluded from the multivariate analysis. So, all variables with an expected cell <5 have been excluded from the multivariate analysis. Odds ratios and p values for factors that remained significant or not significant after controlling for other significant on univariate analysis factors are presented in Tables 2 and 3.

**Table 2:** Multivariate analysis of significant factors in sleeping environment for risk of sudden infant syndrome

Variables*	**Univariate**		**Multivariate**			
			
			**Sleeping+**		**All factors++**	
	
	**Odds ratio (95% CI)**	**p**	**Odds ratio (95% CI)**	**p**	**Odds ratio (95% CI)**	**p**
Sleeping in a bassinet	8.95 [3.80; 21.05]	<0.001	6.54 [2.45; 17.42]	<0.001	4.22 [1.16; 15.38]	0.029
Substandard infant mattress for sleep**	3.09 [1.38; 6.89]	0.006	3.36 [1.27; 8.84]	0.014	2.25 [0.69; 7.34]	0.180
Waterproof cloth over the mattress	2.35 [1.11; 4.98]	0.025	3.83 [1.43; 10.25]	0.007	2.87 [0.81; 10.21]	0.103
≥4 togs during sleep	8.02 [3.55; 18.16]	<0.001	7.93 [3.16; 19.90]	<0.001	8.49 [2.38; 30.32]	0.001

*Variables are for routine practices.

**Includes pallet, twisted plaid, pillow

^+ ^Controlled for all sleeping environment factors listed in table.

^++ ^Controlled for all factors that remained significant in the univariate analysis, including factors of sleeping environment.

**Table 3:** Multivariate analysis of significant factors in background characteristics for risk of sudden infant syndrome

**Variables**	**Univariate**		**Multivariate**			
			
			**Background+**		**All factors++**	
	
	**Odds ratio (95% CI)**	**p**	**Odds ratio (95% CI)**	**p**	**Odds ratio (95% CI)**	**p**
Bottle feeding of the birth	4.89 [1.59; 15.05]	0.006	2.74 [0.45; 16.75]	0.274	2.43 [0.17; 33.74]	0.509
Unplanned pregnancy	8.75 [3.21; 23.85]	<0.001	6.98 [2.19; 22.24]	0.001	5.22 [1.49; 18.18]	0.009
Late (month 4–9) or no prenatal care	4.25 [1.91; 9.41]	<0.001	3.65 [1.24; 10.71]	0.019	2.49 [0.73; 8.51]	0.147
≥2 previous live births	2.73 [1.19; 6.24]	0.017	2.01 [0.69; 5.86]	0.199	3.90 [1.00; 15.10]	0.048
Maternal age ≤20 or ≥35 y	3.78 [1.69; 8.41]	0.001	1.98 [0.67; 5.89]	0.216	1.18 [0.34; 4.04]	0.792
Maternal education ≤12 y	5.93 [2.62; 13.40]	<0.001	4.11 [1.48; 11.41]	0.007	4.48 [1.34; 14.94]	0.015
Single mother	3.04 [1.09; 8.54]	0.034	1.86 [0.36; 9.61]	0.457	1.87 [0.22; 16.06]	0.566
No waged income	3.15 [1.24; 8.02]	0.016	1.01 [0.26; 3.86]	0.993	1.04 [0.21; 5.10]	0.965
Exposure to smoking during pregnancy (one or both of parents smoked)	3.94 [1.62; 9.61]	0.003	2.76 [0.87; 8.71]	0.083	1.87 [0.51; 6.94]	0.348

^+ ^Controlled for all background factors listed in table.

^++ ^Controlled for all factors that remained significant in the univariate analysis, including background factors.

Table 2 shows how the significance of the variables associated with the sleeping environment changes when they are put in the multivariate model with each other and how it changes further when we control for other significant risk factors. Table 3 shows how the significance of the background variables associated with the infant and maternal medical history, parental socioeconomic status and lifestyle changes when they are put in the multivariate model with each other and how it changes further when we control for other significant risk factors. The risk associated with routine sleeping in a bassinet, routinely practiced heavy wrapping (≥4 togs) of an infant during sleep, unplanned pregnancy, 2 or more previous live births and maternal education ≤12 years remained significant when we controlled for all factors. The risks associated with a substandard infant mattress and a waterproof cloth over the mattress for sleep were significant among the factors associated with the sleeping environment but just failed to reach significance when we controlled for all risk factors. The risk associated with a late (month 4–9) or absent prenatal care was significant among the background factors but failed to reach significance when we controlled for all risk factors. The risks associated with a bottle feeding of the birth, maternal age ≤20 y or ≥35 y, single mother, households having no waged income and exposure of a fetus to smoking during pregnancy failed to reach significance both when we controlled for background and for all risk factors.

Later on, models were constructed with the backward stepwise procedure for variables significant at the 5% level. The multivariate model with the best prognostic power of multivariate logistic regression analysis including all effect modifiers that were significant in the univariate analysis and remained significant in the multivariate analysis was produced on the 8^th ^step (Table 4). Therefore, we have established that the variables, such as unplanned pregnancy, routine use of a waterproof cloth over the mattress for sleep, routinely practiced heavy wrapping (≥4 togs) of an infant during sleep, maternal education ≤12 years, 2 or more previous live births and routine sleeping in a bassinet being together significantly predict SIDS victim.

**Table 4:** Associations of variables in the multivariate model with the best prognostic power (on step 8^th^)

**Variables**	**Odds ratio (95% CI)**	**p**
Unplanned pregnancy	6.22 [1.93; 20.07]	0.002
Waterproof cloth over the mattress*	2.65 [0.87; 10.35]	0.101
≥4 togs during sleep*	9.51 [2.91; 31.05]	<0.001
Maternal education ≤12 y	5.97 [1.98; 17.96]	0.001
≥2 previous live births	5.38 [1.52; 19.06]	0.009
Sleeping in a bassinet*	5.14 [1.61; 16.36]	0.006

*Variables are for routine practices.

## Discussion

Till now no observational analytic study concerning SIDS had been performed in Lithuania and data about SIDS were fragmental. Our study has disclosed some specific risk factors significantly associated with and predicting increased risk of SIDS relevant in Lithuania. SIDS group of 35 cases was small but sufficient to yield moderate effects with a power of 0.8 for general tests. Low mortality rate from SIDS in Lithuania resulted in a retrospective case-control study. The response rate of both SIDS parents and control parents was high. Matching SIDS victims and controls for the date of birth and region of birth further reduces confounders. For both SIDS cases and controls there was a time lag between the period of the questions related to and the time of the actual questionnaire. We have considered the possibility that some of the associations are the result of a recall bias. However, we believe the effect of a recall bias was minimal as in other retrospective and prospective studies, recall bias has been found not to influence the results. We have considered the possibility of an informational bias too, because of different ways of information collection. However, the effect of informational bias was minimal as during home visits only standardized questions for SIDS cases were asked. Home visits for SIDS cases were made because of low response of cases during other case-control studies regarding deaths in Lithuania.

Seasonality, with an increased incidence during winter and a decreased incidence during summer, has been considered a distinctive feature of SIDS. We have found a higher SIDS incidence in warm season. Before a definitive answer can be given on the role of the seasons, an extensive analysis should be performed on national data, taking into account the seasonality of births and age effects.

Importantly, this investigation has disclosed that prone sleeping position is not a risk factor for SIDS in Lithuania. The average mortality rate from SIDS in Lithuania is 0.3 per 1000 live births and is low, if compared to international mortality rates. Possibly, it is related to rare prone sleeping, prevalence rate of which is not higher than 3–4% [6]. The countries that have achieved prone prevalence rates of 3–10%, following the introduction of risks reducing campaigns, have SIDS mortality rates as low as 0.4–0.5 per 1000 live births [4].

The side sleeping position as a risk factor for SIDS in Lithuania has not been confirmed, either. The side (73%) and back (17%) positions are commonly recommended sleep positions at discharge from maternity unit in Lithuania. Epidemiological studies from England and New Zealand have shown that side sleeping has a slightly higher risk of SIDS than the supine position, though not as great as prone sleeping [7,8]. The higher risk for SIDS among infants placed on their sides may be related to a relative instability of this position. Although infants placed on their sides usually roll to their backs, the risk of rolling to the prone position from the side is significantly greater than rolling to the prone position from the back. Since sleeping on the back is the safest, parents in Lithuania should be prompted to use this position wherever possible.

Other results from the sleep surface analysis were unexpected for us, too. The risk associated with routine sleeping in a bassinet, a substandard infant mattress for sleep and a waterproof cloth over the mattress during sleep were significant among the factors associated with the sleeping environment. Routine sleeping in a bassinet and waterproof cloth over the mattress have entered in the multivariate model with the best prognostic power. Sleeping in bassinets, which are designed mainly for carriage, unlike cots, which are designed for sleep and meet safety standards for infants, may, at least in theory, carry a risk of accidental entrapment and suffocation. As we have found only a low correlation between placing an infant to sleep in a bassinet and households having no waged income, this practice is not a marker for a low economic status, but a stereotype of a routine infant sleeping environment in Lithuania. A waterproof cloth over the mattress during sleep as well as other loose bedding may carry a risk of accidental entrapment and suffocation, too. Epidemiological studies worldwide identified soft surfaces, such as pillows, quilts, comforters, sheepskins and porous mattresses as a significant risk factor, particularly when placed under a sleeping infant [9,10]. However, the risk associated with sleeping on substandard infant mattresses, such as a pallet or a twisted plaid is somewhat disturbing and needs further investigation. So, the findings from sleeping environment analysis are somewhat puzzling, but illustrate the fact of culturally diverse infant care practices.

Some studies investigating SIDS have reported the risk associated with the excessive amount of clothing or bedclothes and high-temperature environments during sleep, particularly for infants lying prone [11]. In our study a routinely practiced heavy wrapping (clothing, caps and socks) of an infant during sleep emerged from a multivariate analysis as one of the most important independent SIDS risk factors, though most infants routinely slept on their sides or supine. The increased SIDS risk associated with overheating is particularly evident when infants sleep prone but is less clear when they sleep supine [11]. As most infants in the present study slept on their sides or supine, adverse effects of heavy wrapping would be less likely. So our findings suggest that interaction between the heavy wrapping of an infant, especially when the head is covered with a cap, and sleeping in a bassinet may exist and relate with a bigger thermal stress.

Some scientific studies have demonstrated that bed sharing can alter sleep patterns of mother and baby [12,13]. These studies have led to speculations that bed sharing may also reduce the risk of SIDS. While bed sharing may have certain benefits such as encouraging breastfeeding there are not studies demonstrating that bed sharing reduces SIDS. Some studies actually suggest that bed sharing under certain conditions such as smoking parents may increase the risk of SIDS [14]. Our study can not confirm or reject these speculations as bed sharing was common only for the controls.

The contribution of artificial feeding to SIDS is not clear and may vary in different communities. On the basis of our results bottle feeding from the birth is not a risk factor for SIDS when controlled for other significant factors on multivariate analysis. However, there are other good reasons to continue promoting breastfeeding.

The higher incidence of cases with low birth weight and preterm birth confirms findings of other studies [15].

Unplanned pregnancy and 2 or more previous live births are significant potentially modifiable risk factors for SIDS found in our study. Although these factors do not point to a specific etiology, they suggest that interventions targeted towards family planning methods may theoretically reduce SIDS incidence in Lithuania.

The data from univariate analysis related with socioeconomic household status, such as parental age at the birth of an infant, parental education, waged income, housing conditions, number of persons living per room and self-perceived parental economic situation have demonstrated it to be significantly lower in SIDS group than in control. Further, the multivariate analysis showed maternal education ≤12 years to be one of the very significant independent SIDS risk factors in Lithuania. Mothers with lower education might care differently for their babies than mothers having higher education, and they may possibly have many worries, other than those about their child. A link between low socioeconomic status and SIDS has been noted in the literature [16].

Maternal smoking during pregnancy is a major, potentially modifiable risk factor found in many other studies [14,17,18]. Epidemiologically it is difficult to distinguish the effect of active maternal smoking during pregnancy from involuntary tobacco smoking so we have analyzed exposure to smoking according to smoking of partners. Even though the exposure to smoking during pregnancy was significant in the univariate model, we found no significant difference in the multivariate model.

The variables found to be significant in case-control study depend on what is included in a multivariate model. Further more extensive and detailed epidemiological research of SIDS is needed in Lithuania in order to understand the complex relationships between these variables and other factors that affect infant health and serve to heighten the risk of SIDS.

## Conclusion

The results of this first population-based case-control study of the SIDS in Lithuania have shed some light on the epidemiology of the syndrome in Lithuania. The circumstance that some of these factors may be modifiable has important implications in terms of social policy and health education. Although the mortality of SIDS in Lithuania is not high, it might be lowered moreover by public informing about SIDS and related risk factors. Special attention must be paid to mothers with low education on potentially modifiable risk factors such as routine heavy wrapping of an infant during sleep, routine sleeping in a bassinet and unplanned pregnancy.

## Competing interests

The author(s) declare that they have no competing interests.

## Authors' contributions

The study was conceived and designed by Vilija Bubnaitienė and Ramunė Kalėdienė with assistance from Rimantas Kėvalas. The study was conducted under the supervision of Ramunė Kalėdienė. Statistical analysis was conducted by Vilija Bubnaitienė with assistance from Ramunė Kalėdienė and Irena Nedzelskienė. Interpretation of results was conducted by Vilija Bubnaitienė with assistance from Ramunė Kalėdienė. The manuscript was prepared by Vilija Bubnaitienė and edited by Ramunė Kalėdienė and Rimantas Kėvalas. All authors read and approved the final manuscript.

## Pre-publication history

The pre-publication history for this paper can be accessed here:


